# Fertility counseling and preservation considerations in patients with gynecologic and non‐gynecologic *BRCA* and Lynch‐associated malignancies

**DOI:** 10.1002/ijgo.71419

**Published:** 2026-10-09

**Authors:** Anna Weimer, Nadeem Abu‐Rustum, David Mutch, Mauricio Cuello, Angélica Fletcher, Hextan Y. S. Ngan, Nozomu Yanaihara, Rene Pareja, Christina Fotopoulou, Kenneth Ruzindana, Ka Yu Tse, Yana Zou, Neerja Bhatla, Ting‐Chang Chang, Munanchiso Ndukwe Iheme, Jalid Sehouli, Nicole Conchin, Eva Chalas, Sarikapan Wilailak, Barbara Schmalfeldt, Alexander B. Olawaiye

**Affiliations:** ^1^ Department of Obstetrics, Gynecology and Reproductive Sciences University of Pittsburgh School of Medicine Pittsburgh Pennsylvania USA; ^2^ Department of Surgery Memorial Sloan Kettering Cancer Center New York New York USA; ^3^ Department of Obstetrics and Gynecology Washington University School of Medicine St. Louis Missouri USA; ^4^ Department of Gynecology Pontificia Universidad Católica de Chile Santiago Chile; ^5^ Department of Gynecologic Oncology Instituto Nacional de Cancerología Bogotá Colombia; ^6^ Department of Obstetrics and Gynecology The University of Hong Kong Hong Kong Hong Kong; ^7^ Department of Obstetrics and Gynecology The Jikei University School of Medicine Tokyo Japan; ^8^ Department of Surgery and Cancer Imperial College London UK; ^9^ Department of Obstetrics and Gynecology University of Rwanda Kigali Rwanda; ^10^ Department of Obstetrics and Gynecology Shenyang Agricultural University Shenyang China; ^11^ Department of Obstetrics and Gynecology All India Institute of Medical Sciences New Delhi India; ^12^ Department of Obstetrics and Gynecology Chang Gung University College of Medicine Taoyuan Taiwan; ^13^ Department of Gynecology and Obstetrics University Hospital Hradec Králové Hradec Králové Czech Republic; ^14^ Department of Gynecology with Center for Oncological Surgery Medical University of Berlin Berlin Germany; ^15^ Department of Obstetrics and Gynecology Medical University of Vienna Vienna Austria; ^16^ New York University Grossman Long Island School of Medicine Long Island New York USA; ^17^ Faculty of Medicine Ramathibodi Hospital Mahidol University Bangkok Thailand; ^18^ Department of Gynecology University Medical Center Hamburg‐Eppendorf Hamburg Germany

**Keywords:** *BRCA* mutation, cancer prevention, cancer therapy, fertility preservation, Lynch syndrome, malignancies

## Abstract

Lynch syndrome and *BRCA*‐mutation associated malignancies are fortunately rare. Affected patients have an increased risk of cancer diagnosis at a young age, often significantly overlapping with their reproductive years. In helping these patients, oncologists must navigate the following areas of care: cancer prevention, impact of cancer prevention strategies on fertility, impact of cancer treatment on fertility, effective cancer therapies, and preservation of fertility without compromising oncologic outcomes. This intersectionality between fertility preservation and effective cancer prevention/therapy makes the care of these patients extremely complex. With the utility of multidisciplinary care models, careful employment of evolving new treatment strategies, it is possible for patients to achieve their fertility desires while at the same time being protected from the risks imposed by their genetic mutations.

## 1 INTRODUCTION

Ten percent of cancers are caused by hereditary cancer syndromes, the most common of which are hereditary breast and ovarian cancer syndrome and Lynch syndrome.^1^ Both are autosomal dominant conditions that are caused by a pathogenic variant in the *BRCA1*/*2* and the family of mismatch repair (MMR) genes, respectively. Although associated with other malignancies, pathogenic variants of *BRCA1/2* are most commonly associated with breast and ovarian cancer, while Lynch syndrome increases the risk of primarily epithelial colorectal, endometrial, and ovarian cancers.^1^ Both gene families play a central role in maintaining the fidelity of DNA replication and repair.

Those with hereditary cancer syndromes commonly experience cancer diagnoses at earlier ages as their predisposed germline mutations are associated with an impaired capacity to repair DNA damage, resulting in accelerated accumulation of additional genomic abnormalities. On average, *BRCA1/2* mutational carriers are diagnosed with ovarian cancer 10 years before the average age at diagnosis and breast cancer risk increases most rapidly between 31 and 40 years of age.^2^, ^3^ Similarly, patients with Lynch syndrome are diagnosed with endometrial cancer 10 years earlier than their counterparts and their average age at the time of colorectal cancer (CRC) diagnosis is 45 years.^4^ Due to the younger age of diagnosis, there is a disproportionate population of premenopausal patients who may not have completed desired childbearing navigating complex decisions about their fertility and oncologic treatment.

Fertility counseling among cancer patients is nuanced. It requires an understanding of the multifocal impacts of cancer‐directed therapy on future fertility potential as well as a balanced conversation about the risks of delaying treatment while pursuing fertility preservation, if desired. Counseling of these patients is made more challenging by the presence of a hereditary oncogenic mutation, such as *BRCA1/2* pathogenic variants or Lynch syndrome, as there is need for further risk stratification at each step of the fertility preservation conversation. Much of the literature related to fertility counseling for *BRCA1/2* and Lynch carriers focuses on navigating decisions surrounding fertility preservation before prophylactic oophorectomy or hysterectomy, rather than those with an incident cancer diagnosis, even though the group with incident cancer diagnosis remain extremely important to clinical considerations. Furthermore, the literature for those with incident cancer is heavily focused on breast cancer.

Here, we provide a narrative review of the fertility counseling needs for *BRCA1/2* mutations or Lynch syndrome carriers diagnosed with an associated gynecologic or non‐gynecologic cancer, including breast cancer and CRCs. Although there are other genes, such as *RAD51C* and RAD51*D*, that produce a similar phenotype and cancer risk profile to *BRCA1/2* mutations, we focus on *BRCA1/2* mutations specifically given more robust data. We focus on the fertility impacts of oncologic treatment approaches, including new and emerging agents, options for fertility preservation and their associated efficacy and oncologic safety in the carrier population when used during and after cancer diagnosis, and safety of pregnancy itself after diagnosis.

## 2 METHODS

This narrative review examined the current evidence on fertility preservation, reproductive outcomes, and pregnancy considerations in patients with hereditary cancer syndromes, particularly *BRCA1/2* pathogenic variants and Lynch syndrome, undergoing treatment for gynecologic and non‐gynecologic malignancies. This was not a systematic review, and no formal quality appraisal of articles was applied. Relevant literature published in English through January 2026 was identified through searches of PubMed/MEDLINE, Scopus, and Google Scholar. Search terms included combinations of keywords and MeSH terms related to hereditary cancer syndromes, fertility preservation, assisted reproductive technology (ART), pregnancy after cancer, poly ADP‐ribose polymerase (PARP) inhibitors, immunotherapy, and associated malignancies such as breast, ovarian, endometrial, and colorectal cancers. Reference lists of relevant articles and international guidelines were also manually reviewed to identify additional studies.

Included publications comprised clinical trials, observational studies, systematic reviews, meta‐analyses, society guidelines, and selected case reports addressing fertility preservation and reproductive outcomes in hereditary cancer populations. Studies focusing solely on non‐hereditary malignancies, male fertility, or unrelated survivorship outcomes were generally excluded unless clinically informative. Greater emphasis was placed on recent and higher‐quality evidence where available. Due to heterogeneity in study design and limited prospective data, findings were synthesized using a qualitative narrative approach rather than a formal meta‐analysis.

## 3 IMPACTS OF CANCER‐DIRECTED THERAPIES ON FERTILITY FOR GYNECOLOGIC HEREDITARY MALIGNANCIES

The surgical management of gynecological cancers associated with *BRCA1/2* mutations and Lynch syndrome have direct impacts on fertility. We explore the available data on the use of fertility‐sparing surgical interventions for patients' diagnoses with hereditary endometrial and ovarian cancer to guide patient counseling. Treatment of these malignancies can include chemotherapy, immunotherapy, and radiation, the impacts of which are described further when exploring treatment of non‐gynecologic malignancies.

### 3.1 Endometrial cancer

Hysterectomy, typically with bilateral salpingo‐oophorectomy with the appropriate lymph node and peritoneal staging depending on the histological and molecular subtype, is the standard of care management of presumed early endometrial cancer. Although not considered standard of care, the National Comprehensive Cancer Network provides guidelines for the consideration of fertility‐sparing management of uterine‐confined, non‐invasive (stage IA), grade 1 endometrioid endometrial carcinomas with progestin‐based therapy with or without hysteroscopic resection, followed by routine endometrial sampling surveillance, and immediate attempts at pregnancy once remission is achieved. Hysterectomy is then usually recommended at completion of successful pregnancy.^5^ The global complete response rates (defined as negative biopsy control) after either oral or intrauterine device progestins are in the range of 66%–86%, with a higher response rate achieved when medical treatment is combined with hysteroscopic resection; nevertheless, the recurrence rate is still high, in the range of 11%–30%.^6^, ^7^ Progestins are effective as they counter the impacts of unopposed estrogen, a classic driver of endometrial hyperplasia and carcinogenesis in endometrial cancer. However, there is currently no consensus as to the safety of fertility‐sparing approaches for Lynch‐associated endometrial cancers due to concerns about decreased responsiveness and resistance to standard progestin‐based therapies and increased recurrence rates among the MMR‐deficient (dMMR) population compared to MMR proficient cancers.^8^, ^9^, ^10^, ^11^ Lynch syndrome is not considered an absolute contraindication to conservative management, but patients should be counseled appropriately about the increased risk of recurrence and progression and be connected to a fertility specialist at the time of diagnosis for optimal outcomes.^12^

Due to the decreased response to progestins among dMMR tumors and known alternative molecular driver of carcinogenesis, there has been growing interest in the use of immunotherapy for fertility‐sparing treatment of Lynch‐associated endometrial cancers given their proven efficacy in the treatment of this molecular subtype. Yang et al.^13^ described four cases of stage IA–IIA Lynch‐associated endometrial cancers that were able to achieve a complete response with immunotherapy without recurrence during 3–41 months of follow‐up and with one live birth. The authors suggest that given these promising outcomes, immunotherapy may be a reasonable alternative to progestins for Lynch‐associated endometrial cancer and may potentially be used safely outside the more restrictive criteria used for consideration of fertility‐sparing management; however, further investigation with prospective data in larger cohorts is needed. Two clinical trials (NCT06549855 and NCT04046185) are planned to further investigate the efficacy of combination progestin and immunotherapy for medical management of dMMR endometrial cancer but are yet to start recruiting.

### 3.2 Ovarian cancer

Similarly, ovarian cancer management commonly involves radical surgery, including removal of both ovaries, fallopian tubes, and uterus in addition to a complete staging and/or cytoreductive procedure, depending on the extent of the disease, leaving patients infertile. Although there are recommendations available for fertility‐sparing management of early‐stage epithelial ovarian cancer, with a favorable histologic subtype, with unilateral salpingo‐ophorectomy and uterine preservation or uterine preservation alone after oocyte retrieval, their use in *BRCA1/2* or Lynch mutational carriers is debated and, some argue, contraindicated.^14^ The most recent fertility‐sparing European guidelines consider only stage IA a favorable oncological group for preserving fertility, with stages IC1 and IC2 in a gray zone because of insufficient or conflicting data. The guideline indicates that patients with high‐risk genetic predisposition should have similar fertility‐preservation counseling compared with non‐carriers, but the level of evidence is low.^15^ Large database studies, systemic reviews, and meta‐analyses support favorable oncologic and fertility outcomes with conservative surgery, but none include data specifically on the population affected by *BRCA*/Lynch mutations, nor do they report rates of hereditary mutational carriers in the populations studied.^16^, ^17^, ^18^ There has been a case report of a successful pregnancy after conservative management of fallopian tube cancer in a *BRCA2* carrier;^19^ however, large‐scale safety data are not available to advise routine use. There are no data available in Lynch‐associated ovarian cancers. Any conservative surgical intervention offered should be done in coordination with multidisciplinary care and only after appropriate discussion with the patient of unknown associated risks.

## 4 IMPACTS OF CANCER DIRECTED THERAPY ON FERTILITY FOR NON‐GYNECOLOGIC HEREDITARY CANCERS

*BRCA1/2* and Lynch carriers with associated hereditary malignancies are exposed to a variety of treatments that can have a detrimental impact on their fertility. Here we explore the impacts of cytotoxic chemotherapy, new and emerging chemotherapeutic agents, surgery, and radiation on future fertility among hereditary breast cancer and CRC, the two most common non‐gynecologic malignancies diagnosed in the *BRCA1/2* and Lynch population, respectively.

### 4.1 Impact of medical management on fertility

#### 4.1.1 Cytotoxic agents

Cytotoxic chemotherapy may be indicated in the management of both breast cancer and CRC based on stage and pathologic disease features. Cytotoxic chemotherapies are associated with the risk of treatment‐induced premature ovarian failure and associated infertility from multiple mechanisms including the following: (1) direct cytotoxic effects on the gonads through DNA damage, particularly to ovarian primordial follicles; (2) insults to the hypothalamic–pituitary‐gonadal (HPG) axis; and (3) systemic sequalae of undergoing therapy.^20^ A broader discussion of the biologic underpinnings of these effects is outside the scope of this review.

Impacts of cytotoxic agents on fertility vary by drug, combination therapy, dose, and patient age. Breast cancer therapy typically involves multidrug regimens including a combination of alkylating agents, anthracyclines, taxanes, and antimetabolites, which carry an intermediate (20%–80%) to low risk (<20%) of premature ovarian failure based on the exact regimen used and age of the patient.^21^ Older women are more likely to experience infertility after chemotherapy due to age‐related ovarian changes, including decreased quality of primordial follicles, reduced quantity at baseline, and subsequent increased detriment with chemotherapy‐related follicular loss. Half of breast cancer patients aged over 40 years would experience irreversible ovarian failure after cytotoxic therapy compared to only 15%–30% of women aged under 35 years.^22^

CRC chemotherapy regimens commonly include fluorouracil, oxaliplatin, capecitabine, and irinotecan. Fluorouracil, oxaliplatin, and irinotecan are all potentially gonadotoxic.^23^ Retrospective studies have demonstrated relatively low rates of persistent amenorrhea with fluorouracil and oxaliplatin, in the range of 16%–22%.^24^, ^25^

*BRCA1/2* carriers carry an additional theoretic concern for increased risk of cytotoxic effects of chemotherapy on ovarian tissue due to the impaired biological function of *BRCA 1* and 2 proteins. *BRCA 1* and *2* are involved in the repair of double‐stranded DNA breaks; therefore, deficient tissue, including ovarian tissue, may not be optimally suited to repairing the chemotherapy‐induced damage, leaving tissue more prone to damage than those in the general population. However, studies are mixed. In a prospective analysis, germline *BRCA* mutations have been shown to be a predictor of persistent amenorrhea at 18 months after breast cancer treatment (odds ratio 5.59; *P* = 0.03).^26^ However, studies examining ovarian tissue toxicity in patients with *BRCA1*‐mutated and wild‐type breast cancer found no difference in the ovarian toxicity observed in vitro or in vivo.^27^ Furthermore, additional studies in humans and mice have failed to demonstrate an additive deleterious gonadotoxic effect of carrying a *BRCA* mutation beyond the baseline negative impact of cytotoxic chemotherapy.^28^, ^29^

Similar concerns exist for patients affected by Lynch syndrome. The MMR proteins contribute to meiosis, genomic stability, and folliculogenesis through repair of double‐stranded DNA breaks during crossover. Pathogenic variants of MMR genes are associated with premature ovarian aging and decreased fertility in mouse models,^30^ suggesting that MMR‐mutated ovarian tissue may be subject to inadequate repair mechanisms and therefore be more sensitive to cytotoxic chemotherapy. However, data in humans are less clear and no studies have demonstrated an increased risk of premature ovarian failure with cytotoxic chemotherapy in Lynch syndrome carriers compared to the general population.^31^ Theoretical concerns should be discussed with both *BRCA1/2* and Lynch syndrome carriers, but further data are needed before any definitive conclusions can be drawn and the patient should be offered fertility preservation before initiation of cytotoxic therapy, if feasible.

Strategies to limit ovarian toxicity are promising. Temporary ovarian suppression with gonadotropin‐releasing hormone agonists (GnRHa) during chemotherapy should be offered to patients with breast cancer desiring future fertility based on the results of the clinical trials PROMIS, POEMS‐SWOG S0230, and OPTION, which demonstrated a reduction in treatment‐related premature ovarian failure and increased pregnancy rates with use.^32^, ^33^, ^34^ No studies have examined GnRHa use during treatment for CRC.^35^ Despite potential benefits in limiting ovarian toxicity, the American Society of Clinical Oncology cautions against the use of GnRHa in place of establish fertility‐preserving methods such as oocyte or ovarian cryopreservation.^36^

#### 4.1.2 Endocrine therapy

Endocrine therapy may be indicated as adjuvant therapy in the management of breast cancer. Tamoxifen, a selective estrogen receptor modulator, is the most commonly used agent in premenopausal patients with breast cancer with suggested use for up to 10 years. Although tamoxifen has not been shown to impact ovarian reserve or ovarian aging, it can induce amenorrhea; moreover, the need for prolonged treatment can overlap significantly with years of reproductive potential, thus limiting fertility.^20^ Fortunately, prospective studies have demonstrated high rates of menstrual resumption and favorable oncologic outcomes with temporary interruption of endocrine therapy to attempt pregnancy.^26^ Patients should be counseled about the option of temporarily interrupting treatment, particularly in combination with ART to minimize time off therapy.

#### 4.1.3 PARP inhibitors

PARPs play an essential role in the detection and repair of DNA damage, making them a useful target for chemotherapeutics, particularly among *BRCA1/2* carriers with breast or ovarian cancer given the synergistic benefit in cells already deficient in homologous recombination DNA repair.^37^ Although PARP inhibitors (PARPi) are only currently indicated in advanced ovarian cancer where fertility preservation is not recommended, they are indicated in advanced or metastatic breast cancer.^38^ Owing to PARPs' known role in the production of oocytes and maintenance of chromosomal stability in meiosis, there are concerns about potential implications for fertility in PARPi‐exposed women.^39^ Although minimal clinical data exist, preclinical studies have demonstrated several potential negative impacts of PARPi in mouse models, including significant depletion of the ovarian reserve, decreased quality and function of granulosa cells, and decrease oocyte retrieval among PARPi‐exposed mice.^40^, ^41^ Notably, in contrast to murine data, a recent in vitro study using human ovarian tissue found no evidence of olaparib‐ or niraparib‐induced primordial follicle depletion or granulosa cell dysfunction,^42^ highlighting the discordance between preclinical and human models and the need for prospective clinical studies measuring ovarian reserve before and after PARPi exposure in women of reproductive age. There is also ongoing concern among *BRCA1/2* carriers that ovarian depletion may be compounded with baseline deficiencies in DNA repair, leading to a greater impact on meiosis and increased DNA damage accumulation and oocyte decline.^39^, ^43^ Counseling should include conversation about these potential risks.

#### 4.1.4 Immunotherapy

Immunotherapy agents are increasingly being used across the cancer landscape. These agents are particularly effective in dMMR tumors and, as such, Lynch syndrome carriers with CRC are at increased risk of exposure to them. Immunotherapy may also be used in Lynch‐associated endometrial cancer but is currently only indicated in advanced stages. *BRCA1/2* carriers may also have exposure to immunotherapy for the treatment of triple‐negative breast cancer. As this class of medications expands in both indication and prevalence of use, the potential impact on future fertility is of growing concern.^43^

Potential deleterious impacts on fertility have been studied in vitro and in animal models, with studies demonstrating an altered ovarian immune microenvironment leading to primordial follicle depletion and impaired ovulation.^44^, ^45^ The implications of these findings in humans are not yet known but there are case reports of successful pregnancies and no increased risk of premature ovarian failure after exposure to checkpoint inhibitors.^46^, ^47^ Furthermore, the impact of immunotherapy on ovarian reserve as measured by anti‐Mullerian hormone (AMH) is inconsistent across studies.^48^, ^49^ A prospective sub‐analysis of the GeparDouze/NSABP B‐59 trial—the first prospective human data on this question—demonstrated that AMH fell to undetectable levels at the end of neoadjuvant chemoimmunotherapy with atezolizumab without recovery during follow‐up, with numerically higher rates of chemotherapy‐induced ovarian failure compared to chemotherapy alone.^50^ Whether this effect is attributable to immunotherapy, chemotherapy, or their combination remains to be established. Further prospective studies are needed, particularly with PD‐1 inhibitors used in dMMR Lynch‐associated and BRCA‐associated malignancies.

The toxicities of immunotherapy, including hypothyroidism and hypophysitis,^51^ may also impact fertility by disrupting the HPG axis, causing ovulatory dysfunction and increasing the risk of miscarriage.^52^ Patients experiencing challenges with fertility after immunotherapy should be screened for hormonal causes.

### 4.2 Impact of bowel surgeries on fertility

Less often discussed and possibly underrecognized is the impact of bowel surgery on future fertility. Surgery is the cornerstone of CRC management and many patients with Lynch‐associated bowel malignancy will undergo an indicated bowel resection. The impact of bowel surgery on fertility in CRC is poorly understood. However, in benign colorectal pathologies, including ulcerative colitis and familial adenomatous polyposis, surgical management, particularly involving resection below the peritoneal reflection, appears to have a greater impact on fertility compared to medical management. This is thought to be driven by adhesive disease in combination with damage to pelvic nerves and reproductive organs with lower pelvic dissections.^53^ A limited series suggests there may be a potential reduction of the impact on fertility with the use of minimally invasive techniques, but more robust data are needed to draw conclusions.^54^ Furthermore, studies specifically related to cancer‐directed surgical outcomes are needed to further understand the associated fertility risks for these patients.

Surgical‐sparing techniques are also employed as immunotherapy is emerging as a potential medical treatment strategy for early‐stage dMMR CRC, based on promising survival data from both prospective trials and systematic reviews.^55^, ^56^, ^57^ If sustained complete responses to immunotherapy permit deferral or avoidance of surgery in selected patients, this may represent a fertility‐preserving strategy analogous to the emerging use of immune checkpoint inhibitors in Lynch‐associated endometrial cancer. To date, however, no published data specifically examine surgery‐avoidance rates, fertility outcomes, or pregnancy rates after immunotherapy in Lynch syndrome–associated CRC. Further, there are no comparisons in fertility outcomes between CRC treated with surgery versus immunotherapy, given the potential fertility impacts of both modalities.

### 4.3 Impact of radiation

Lynch‐associated CRC can largely be managed by surgical resection with or without adjuvant chemotherapy/immunotherapy; however, there is a potential indication for pelvic radiation in the treatment algorithm for selected cases of surgically unresectable or metastatic disease, or for rectal carcinomas. Studies suggest that over 90% of patients undergoing full‐dose pelvic therapy (45–50 Gy) for rectal carcinoma would experience premature ovarian failure.^35^ Ovarian preservation strategies should therefore be pre‐emptively offered to patients for whom pelvic radiation is indicated. Such strategies can include pre‐radiation oocyte or embryo preservation and ovarian transposition with or without uterine transposition or fixation to a location outside of the radiation field.^58^ Meta‐analysis has demonstrated that ovarian transposition led to a pooled incidence of 66.9% preserved ovarian function among 133 patients across 10 studies.^59^ Further studies are needed to explore fertility outcomes among this population as data are limited.

Although the uterus is less radiosensitive than ovarian tissues, pelvic radiation does impact endometrial function, uterine volume and elasticity, and uterine vascular supply, all of which have potential negative effects on future pregnancy.^60^ Uterine transposition is less well studied than ovarian transposition but is emerging as a fertility‐sparing technique for patients undergoing pelvic radiation. Data are largely limited to case series, but a 2023 review of cases reported to date (n = 18) demonstrated the feasibility of the technique, with successful uterine preservation and minimal complications; three of five patients attempting pregnancy achieved spontaneous pregnancies and live births.^61^ Larger cohorts are needed to fully understand feasibility and obstetric outcomes and will likely require multicenter collaboration, given the limited number of cases. Furthermore, no data exist specifically for patients with Lynch syndrome, and given the risk of endometrial cancer, this technique should be considered with caution.

Although *BRCA*‐associated breast cancer patients may undergo radiation therapy, such treatments are generally outside the pelvis and therefore have little or no impact on fertility.

## 5 FERTILITY PRESERVATION AND ASSISTED REPRODUCTIVE TECHNOLOGY

Before the initiation of potentially gonadotoxic therapy, patients interested in fertility preservation should have the opportunity to meet with an oncofertility specialist to discuss available options. These options include oocyte, embryo, and ovarian tissue cryopreservation; however, details of the required procedures are outside the scope of this review. All conversations about available fertility‐preservation options should involve multidisciplinary discussion between oncology providers, fertility specialists, and patients to ensure a comprehensive assessment of the risks of delaying treatment and the fertility‐preservation options available to each individual patient. A summary of the available evidence and counseling recommendations across hereditary syndromes, cancer types, and treatment modalities is provided in Figure 1. Assuming that it is deemed safe to proceed with any of the fertility‐preservation options, we will now focus on relevant counseling specific to patients with *BRCA1/2* germline mutations and Lynch syndrome–associated gynecologic, breast, or CRC, including the safety of the procedures in terms of oncologic outcomes, the efficacy of oocyte retrieval in the carrier population, and the availability of pre‐implantation genetic testing in the case of embryo cryopreservation.

**FIGURE 1:**
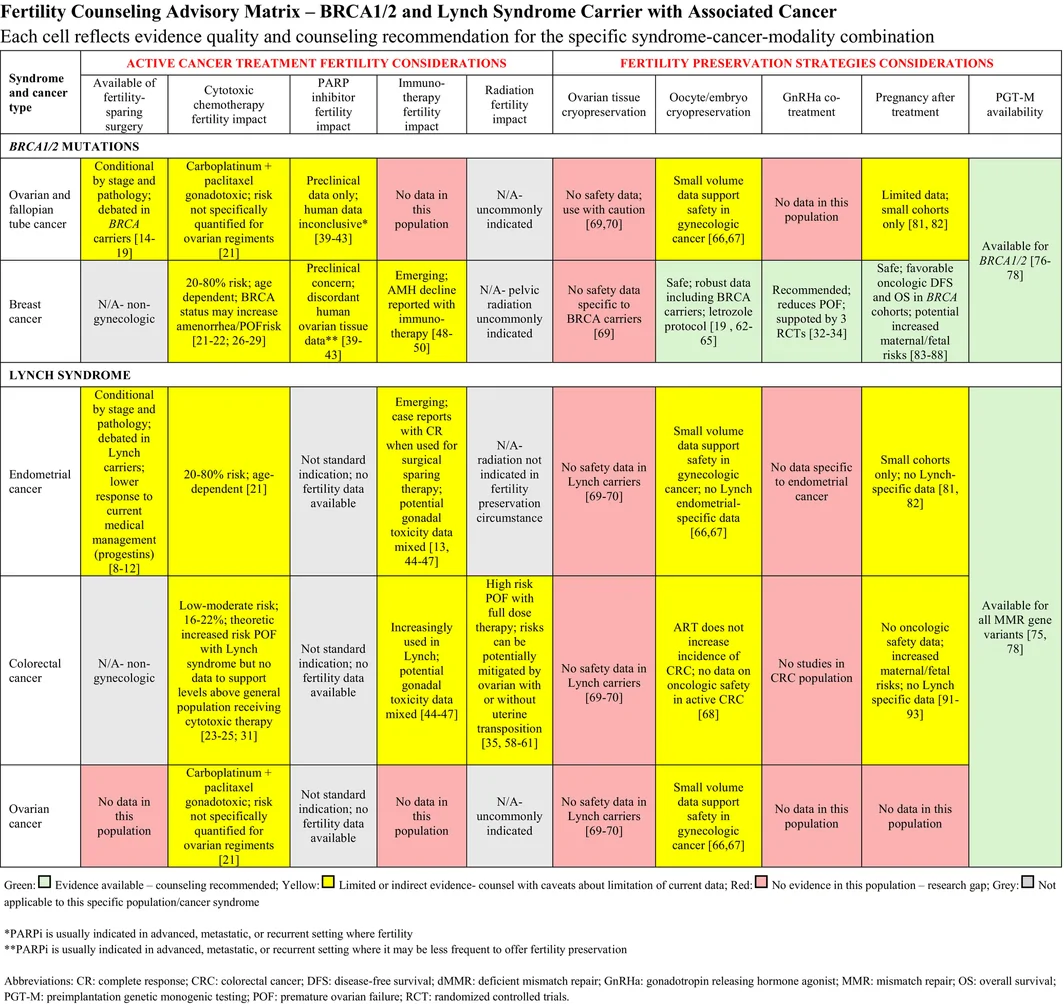
Fertility counseling advisory matrix for *BRCA1/2* and Lynch syndrome carriers with active cancer diagnosis. Each cell reflects the quality of available evidence and the corresponding counseling recommendation for the specific combination of hereditary syndrome, cancer type, and treatment modality. Green cells indicate that evidence is available and counseling is recommended, based on population‐specific or strong indirect data. Yellow cells indicate limited, indirect, or extrapolated evidence; counseling is appropriate but should be accompanied by explicit caveats. Red cells indicate no evidence in this specific population and identify priority areas for future research. Columns represent 10 clinical decision points: (A) fertility‐sparing surgery, (B) cytotoxic chemotherapy gonadotoxicity, (C) PARPi fertility impact, (D) immune checkpoint inhibitor fertility impact, (E) radiation fertility impact, (F) ovarian tissue cryopreservation safety, (G) oocyte and embryo cryopreservation safety, (H) GnRH agonist co‐treatment, (I) safety of pregnancy after treatment, and (J) pre‐implantation genetic monogenic testing availability. AMH, anti‐Mullerian hormone; ART, assisted reproductive technology; CR, complete response; CRC, colorectal cancer; DFS, disease‐free survival; dMMR, deficient mismatch repair; GnRHa, gonadotropin‐releasing hormone agonist; OS, overall survival; PARPi, poly ADP‐ribose polymerase inhibitors; PGT‐M, pre‐implantation genetic testing for monogenic disorders; POF: Premature ovarian failure; RCT, randomized controlled trial. 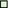 Evidence available – counseling recommended; 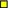 Limited or indirect evidence – counsel with caveats about limitation of current data; 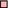 No evidence in this population – research gap; 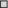 Not applicable to this specific population/cancer syndrome. *PARPi is usually indicated in advanced, metastatic, or recurrent setting where fertility. **PARPi is usually indicated in advanced, metastatic, or recurrent setting where it may be less frequent to offer fertility preservation.

### 5.1 Oncologic safety of fertility‐preservation options

Oocyte and embryo cryopreservation require controlled ovarian follicular stimulation and often lead to supraphysiologic hormonal levels, posing a theoretical risk in hormonally responsive cancers such as breast and gynecologic cancers. This concern is partially mitigated by utilizing letrozole or aromatase inhibitors to control systemic hormonal peak levels, without compromising oocyte yield.^62^

Breast cancer has the most robust safety data for ovarian stimulation. Meta‐analysis has shown no detrimental effect of ovarian stimulation on disease‐free survival (DFS) for breast cancer.^63^ Long‐term follow‐up among breast cancer patients has also demonstrated oncologic safety in patients who underwent ART, including those with hormone‐responsive breast cancer.^64^ Furthermore, among *BRCA*‐mutation carriers, the use of ART after breast cancer diagnosis did not increase the risk of subsequent ovarian cancer^19^ and a case–control study demonstrated no increased risk of breast cancer among those undergoing fertility treatment.^65^ Some small volume data also suggest that ovarian stimulation is safe in gynecologic malignancies, with no demonstrated increase in recurrence rates or mortality.^66^, ^67^ No available studies assessed oncologic outcomes for CRC after fertility preservation; however, studies have demonstrated that the use of ART does not increase the risk of incident CRC.^68^

Ovarian tissue cryopreservation is a fertility‐preservation option for prepubescent girls and can be used when cancer status does not allow time for the 2–5‐week treatment delay needed for ovarian stimulation and oocyte retrieval. However, the use of ovarian cryopreservation and re‐implantation is controversial in *BRCA1/2* mutation and Lynch syndrome carriers, given the risk of ovarian malignancy in transplanted tissue.^69^ This risk can be mitigated by removal of ovarian tissues at completion of childbearing and limiting use of cryopreservation to a younger patient population before the recommended age of prophylactic oophorectomy. However, in the absence of any safety data in this population, it should be used with caution. A recent expert review specifically addressing hereditary cancer syndromes confirmed this gap and recommended that ovarian tissue cryopreservation in *BRCA* and Lynch carriers be considered primarily before risk‐reducing surgery, with mandatory multidisciplinary counseling and consideration of pre‐implantation genetic testing before any future re‐implantation.^70^

### 5.2 Efficacy of oocyte retrieval in the population with BRCA1/2 mutation and Lynch syndrome

Given the biological role of MMR and *BRCA1/2* proteins in meiosis and ovarian function, there are concerns about the potential for decreased ovarian reserve and subsequent lower oocyte yield in *BRCA1/2* mutation and Lynch syndrome carriers undergoing ovarian stimulation. Data, however, are mixed. A recent meta‐analysis assessing fertility‐preservation outcomes in *BRCA* mutation carriers found that there was no difference in the number of oocytes retrieved and no significant difference in antral follicles count; however, there was a decrease in the number of mature oocytes among *BRCA* mutation carriers (median difference − 1.48; *P* = 0.01).^71^ Furthermore, there is inconsistent evidence assessing *BRCA* mutation on AMH levels, a marker of ovarian reserve; however, a meta‐analysis suggests that AMH levels are lower for *BRCA1* mutation carriers but not *BRCA2* mutation carriers.^72^ Overall, the current evidence supports the efficacy of ovarian stimulation in *BRCA1/2* mutation patients with possible decreased oocyte yield, particularly among *BRCA1* mutation carriers.^73^ Counseling should include the potential for impact based on carrier status, and should be individualized based on patients' age, cancer status, and individual follicle count and AMH, as these are more likely to drive oocyte yield than mutation carrier status.

Despite similar concerns about the biological possibility of decreased fertility potential in Lynch syndrome carriers, there are very limited data to understand potential impacts on ovarian stimulation outcomes. Extrapolating from registry data that demonstrated no evidence of premature ovarian failure in the Lynch syndrome population,^31^ ovarian reserve may not be affected by carrier status and, therefore, stimulation outcomes may not be affected; however, no direct information is available to inform counseling.

### 5.3 Pre‐implantation genetic monogenic testing

Among patients undergoing ART with embryo cryobanking, pre‐implantation genetic testing for monogenic disorders (PGT‐M), with selection of genetically unaffected embryos, can be an effective means of preventing transmission to subsequent generations for both *BRCA* mutation and Lynch syndrome.^74^ Survey data among *BRCA1/2* mutation carriers have demonstrated a desire for information and counseling on the availability of PGT‐M, and the majority of patients were accepting of its use despite low personal uptake.^75^, ^76^

PGT‐M, although effective in preventing transmission, incurs additional costs for testing. Although it does not affect the pregnancy outcomes of selected embryos, it has been associated with lower cumulative live birth rates than conventional in vitro fertilization, especially in women with reduced oocyte potential.^77^ Although ART is covered by national insurance schemes in some countries for patients with cancer and mutational carriers, insurance coverage is often inadequate or absent. The need for additional cycles of in vitro fertilization to obtain mutation‐free embryos also limits access to these options for many patients.^78^ Furthermore, the need to undergo additional cycles may delay the initiation of cancer‐directed therapy. To avoid these risks, patients can be offered double stimulation (DuoStim), a protocol used to maximize gamete acquisition in a minimal amount of time.^79^

## 6 SAFETY OF PREGNANCY AFTER CANCER DIAGNOSIS

In order to address safety of pregnancy after a cancer diagnosis, patient counseling should address three fundamental questions: (1) Does a pregnancy after a cancer diagnosis increase the risk of negative oncologic outcomes?; (2) Are there increased pregnancy or fetal risks based on history of prior malignancy and treatment?; and (3) Are there any specific data in a population of *BRCA1/2* mutation or Lynch syndrome carriers?

### 6.1 Gynecologic malignancies

The majority of studies assessing maternal and fetal outcomes in patients with gynecologic cancer tend to focus on incident cancer diagnosed during an established pregnancy. Furthermore, the oncologic safety data for both endometrial and ovarian cancer is largely based on the safety of fertility‐sparing surgery and the associated fertility outcomes in terms of pregnancy and live birth rates alone. Less is reported about any oncologic risks associated with having a pregnancy after cancer diagnosis or maternal and fetal outcomes during subsequent pregnancies, although small cohorts suggest favorable oncological, maternal, and fetal outcomes.^80^, ^81^ No data are available to aid counseling in *BRCA1/2* mutation and Lynch syndrome carriers.

### 6.2 Breast cancer

Despite hormone responsiveness of some breast cancers, pregnancy after breast cancer is considered to be safe for interested patients. A meta‐analysis of oncologic outcomes after pregnancy in hormone‐sensitive breast cancer patients demonstrated no difference in DFS and improved overall survival among those with a subsequent pregnancy compared to those without.^82^ Studies also generally support maternal and fetal safety of pregnancy after breast cancer diagnosis and treatment, albeit with some increased risk.^83^ One meta‐analysis identified an increased risk of cesarean delivery, low birth weight, small for gestational age, and preterm birth in breast cancer survivors.^84^ Data from *BRCA*‐mutation carriers is more limited, but three retrospective *BRCA*‐mutated cohorts have suggested that pregnancy after breast cancer has favorable maternal and fetal outcomes and does not negatively impact DFS or overall survival.^85^, ^86^, ^87^

### 6.3 Colorectal cancer

Although CRC tumors often express estrogen and progestin receptors, the impact of elevated hormone levels during pregnancy has not been established and the data are mixed.^88^ Estrogen has been considered potentially protective against CRC based on differences in the lifetime risk of CRC between men and women.^89^ Lynch syndrome cohorts have demonstrated similarly increased risk in men, with one cohort documenting a risk of CRC by the age of 60 years of 0.77 in men compared with 0.34 in women.^90^ To our knowledge, no studies have examined the impact of pregnancy after CRC diagnosis on oncologic outcomes.

Although pregnancy is considered safe after CRC, as with breast cancer, population data suggest potentially increased maternal and fetal risks among women with a history of CRC. A retrospective national cohort study found that obstetric patients with CRC had an increased risk of death, arrythmia, kidney injury, respiratory failure, severe sepsis, cesarean delivery, preterm birth, and intrauterine death.^91^ Notably, due the nature of the data source, this study did not comment on the temporality of the cancer diagnosis to the pregnancy and therefore these results may reflect increased risk associated with concurrent diagnosis. An Australian study, specifically identifying pregnancy after CRC, found similar increases in maternal and fetal risk, with increased odds of antenatal and postpartum hemorrhage, cesarean delivery, low fetal APGARs, and need for neonatal resuscitation. Risk of maternal and fetal adverse outcomes were also higher for those with rectal compared with colonic malignancy.^92^ A nationwide Swedish case–control study of 207 pregnancies in women with early‐onset CRC independently corroborated these findings, demonstrating 2.5‐fold higher odds of pre‐eclampsia (odds ratio 2.52, 95% confidence interval 1.25–5.08), increased rates of emergency cesarean delivery, and 2.3‐fold higher odds of preterm birth compared to matched controls, with risks again elevated for rectal versus colonic disease.^93^ Collectively, these data support the importance of coordinated obstetric and oncologic management in this population, though none of the available studies include data stratified by Lynch syndrome carrier status.

Further research is warranted to confirm and expand these findings. No data are available specifically for Lynch syndrome–associated CRC aside from case reports. In the absence of further information to guide risk stratification, patients should be counseled about potential increased maternal and fetal risks in addition to coordinated care with appropriate obstetric and oncology specialists for shared decision‐making.

## 7 DISCUSSION

This review highlights the available data to support counseling of patients with incident *BRCA1/2* or Lynch‐associated cancers. It synthesizes and collates data across the spectrum of counseling needs for this patient population, which has been underrecognized in the fertility counseling literature to date and is distinct from asymptomatic carriers. A practical decision‐support reference for clinicians counseling these patients across cancer types and treatment modalities is provided in Figure 1.

However, significant evidence gaps persist that limit the precision of current counseling. Key priorities for future research include the following: prospective studies evaluating the gonadotoxic effects of PARP inhibitors and immune checkpoint inhibitors on ovarian reserve in reproductive‐age women with *BRCA1/2* or Lynch syndrome; registry‐based data on ovarian stimulation outcomes specific to Lynch syndrome carriers; long‐term oncologic safety data for ovarian tissue cryopreservation and re‐implantation in hereditary syndrome carriers; prospective trials of immunotherapy as fertility‐sparing treatment for Lynch‐associated endometrial cancer; and dedicated cohort studies on maternal and fetal outcomes after CRC in Lynch carriers. Future clinical trials enrolling patients with hereditary cancer syndromes should incorporate standardized fertility endpoints to progressively address these gaps.

This review has some limitations, including those inherent in of non‐systematic review processes, which can bias study selection and result in relevant literature being missed. The inclusion of English‐language studies only further limits the literature reviewed. This review also focuses on data from high‐income settings, limiting its applicability to lower‐resource settings, where options for fertility preservation, both surgical and involving ART, are more limited, in part due to the paucity of available literature in this space.

## 8 CONCLUSION

Women of reproductive age affected by *BRCA1/2* mutation and Lynch syndrome–associated cancers can successfully achieve their reproductive aspirations within a multidisciplinary management approach that carefully balances oncologic safety and fertility potential. Vigilant multidisciplinary follow‐up and long‐term coordination are crucial to avoid compromising either aspect. Important evidence gaps remain, highlighting the need for prospective studies and standardized fertility endpoints in this population.

## AUTHOR CONTRIBUTIONS

All authors have made substantial contributions to this work and meet the ICMJE crtieria for authorship.

## CONFLICT OF INTEREST STATEMENT

The authors have no conflicts of interest.

## Data Availability

Data sharing is not applicable to this article as no new data were created or analyzed in this study.
